# The Analgesic Efficacy of Preoperative Oral Ibuprofen and Acetaminophen in Children Undergoing Adenotonsillectomy: A Randomized Clinical Trial

**DOI:** 10.5812/aapm.15049

**Published:** 2014-02-28

**Authors:** Maziar Mahgoobifard, Yalda Mirmesdagh, Farsad Imani, Atabak Najafi, Masoomeh Nataj-Majd

**Affiliations:** 1 Department of Anesthesiology and Children and Adolescent Health Research Center, Ali-Ebne Abitaleeb Hospital, Zahedan University of Medical Sciences, Zahedan, Iran; 2 Heart Value Disease Research Center, Rajaie Cardiovascular Medical and Research Center, Tehran University of Medical Sciences, Tehran, Iran; 3 Department of Anesthesiology, Sina Hospital, Tehran University of Medical Sciences, Tehran, Iran; 4 Research Development Center of Arash Woman's Hospital, Tehran University of Medical Sciences, Tehran, Iran

**Keywords:** Acetaminophen, Ibuprofen, Pain, Postoperative

## Abstract

**Background:**

Adenotonsillectomy is one of the most common surgical procedures in children. Several complications and morbidities are common after nasal surgeries and the most common is pain. Several techniques have been employed to reduce the severity of postoperative pain. One of the preoperative techniques is pre-emptive analgesia through preventive central hypersensitization. This technique is performed by applying analgesic methods before the onset of nociceptive stimuli, consequently decreasing postoperative analgesics requirements.

**Objectives:**

Preoperative oral drug administration for pain analgesia is performed in several methods. The aim of this study was to compare the analgesic effects of preoperatively administration of oral acetaminophen and ibuprofen.

**Patients and Methods:**

In a double-blinded, randomized placebo-controlled study, sixty 4-12 years old ASA I or II children scheduled for elective adenotonsillectomy, were assigned to receive either acetaminophen 15 mg/kg, ibuprofen 10 mg/kg or placebo 30 minutes before the operation. Postoperative pain was assessed using the Children's Hospital of Eastern Ontario Pain Scale (CHEOPS), upon arrival to the post anesthetic care unit (PACU) and ward. Findings were analyzed by SPSS version 17 using variance analysis and Tukey’s test.

**Results:**

The average pain intensities were significantly lower in acetaminophen group based on the CHEOPS in both PACU and ward compared to ibuprofen or placebo groups; but there was no difference in pain intensity between the ibuprofen and placebo groups. Pain intensity in PACU in Acetaminophen group was 7.05 ± 0.64 vs. 8.38 ± 1.20 in placebo group and 8.14 ± 0.85 in ibuprofen group, pain intensity in ward in the acetaminophen group was 6.0.87 ± 0.85in the acetaminophen group, vs. 8.04 ± 1.02 in placebo group, and 7.78 ± 0.78 in ibuprofen group.

**Conclusions:**

This study showed that administration of oral acetaminophen 30 minutes preoperatively, resulted in significantly lower pain intensity in children undergoing adenotonsillectomy in PACU and ward, compared to ibuprofen and placebo.

## 1. Background

Adenotonsillectomy is one of the most common surgical procedures in children (1). Several complications and morbidities are common after nasal surgeries (2). Possible postoperative complications include severe pain, difficulty in swallowing, reduced oral intake, nausea, vomiting and bleeding. These events can negatively affect children’s recovery and increase the length of hospital stay (3). Oral liquid and solid food intake may decrease following the operation due to postoperative pain which may cause sleep disorders and discomfort. Earlier and easier initiation of oral intake, decrease of recovery time and fast return to normal activities are advantages of postoperative pain management (4). Several techniques have been employed to reduce the severity of postoperative pain, including pharmacological methods such as spray forms of Lidocaine in the tonsillar fossa (5) and non-pharmacological protocols (6) like preoperative psycho-educational interventions (7). Some of the pharmacological techniques are applied at the end of operations like intravenous or subcutaneous ketamine (8) or postoperative honey administration (9) and some before operation; administration of opioids and non-opioids (e.g. Pregabalin) (10, 11). One of the preoperative techniques is pre-emptive analgesia through preventive central hypersensitization. This technique is performed by applying analgesic methods before the onset of nociceptive stimuli, consequently decreasing postoperative analgesics requirements (12, 13). Several methods have been developed and studied to manage pain following adenotonsillectomy, including pre-emptive peritonsillar local anesthetic infiltration, pre-incisional opioid use and administration of Paracetamol or non-steroidal anti-inflammatory drugs (NSAIDs) (14, 15).

In some studies, preoperative oral acetaminophen intake (18 mg/kg) was not effective to alleviate pain after myringotomy (16). However, high dose of oral acetaminophen (40 mg/kg) was effective for this matter (17). There are different results regarding the effectiveness of preoperative ibuprofen to provide efficient analgesia (1, 18, 19).

## 2. Objectives

Therefore, in this study we compared the effects of acetaminophen and ibuprofen administration before surgical incision. We compared postoperative pain score following adenotonsillectomy in children given acetaminophen, ibuprofen or placebo.

## 3. Patients and Methods

A total number of sixty patients, aged 4 to 12 years old, in classes I and II of the American Society of Anesthesiologists classification, scheduled for elective adenotonsillectomy in Zahedan Khatam Hospital of in 2011, were enrolled in this prospective randomized clinical trial. An approval was obtained from the Institutional review board of the Zahedan University of Medical Sciences; also, an informed consent was obtained from their parents. The patients were randomly (blocked randomization) divided into 3 groups. Group A (n = 21) received 10 mg/kg oral ibuprofen, group B (n = 18) received 15 mg/kg oral acetaminophen, and group C patients (n = 21) received orally salt tablet as placebo 30 minutes before the operation. Both patients and hospital staff were blinded to the administered analgesics. General anesthesia induction was performed by Fentanyl (2µg/kg), thiopental (5 mg/kg), and atracurium (0.5 mg/kg). Thereafter, all patients were intubated and anesthesia was maintained with sevoflurane and N_2_O /O_2_. No patients in this study received local anesthesia at peritonsillar regions. All patients underwent the same operation procedure, and to decrease the degree of swelling and nausea after the operation all patients received 0.5 mg/kg dexamethasone (maximum of 10 mg, administered intravenously). All patients were extubated before transferring to the PACU. The patients who underwent complicated surgery, prolonged more than 1 hour, were excluded from the study. The Children Hospital Eastern Ontario Pain Scale (CHEOPS) was used to evaluate the pain severity in patients. The scores were assessed by two registered nurses trained how to assess pain using the CHEOPS before the study. The scores were assessed at the time of admission at PACU, 5, 10, 15, 30, 45, and 60 minutes after admission and at discharge from PACU. Finally, the highest score of pain was recorded. To manage the pain in cases with CHEOPS score > 10, with sore throat, 2.5 mg morphine was administered intravenously. The dosage of received morphine was recorded by PACU nurses. All patients were under observation at PACU for 1 hour and then transferred to the ENT ward. All patients were followed in ward, and the CHEOPS score was assessed every 30 minutes for 3 hours. Acetaminophen (10-15 mg) was administered to manage the pain in cases with CHEOPS score > 10 with sore throat at the ward. The mean highestscores of CHEOPS at PACU and ward, mean doses of administered morphine and acetaminophen, mean duration of general anesthesia, operation and PACU stay, and demographic data including age, gender and weight were recorded and analyzed statistically. Results were compared between the groups using one way ANOVA, Chi-square test and Turkey test by using SPSS. P values < 0.05 were considered statistically significant.

## 4. Results

Sixty children were recruited to this study: 21 to group A (ibuprofen syrup), 18 to group B (acetaminophen), and 21 to group C (salt tablet group; control group). There were 33 male and 27 female patients. No significant differences were observed between the three groups regarding age and sex. However, there was a significant difference between the three groups regarding patients' weight. The mean highest scores of pain at PACU were compared between the groups, and a significant difference was detected between the min this criterion (Table 1). The mean highest scores of pain at PACU were also compared between groups A and B, groups C and A and groups C and B, (Table 2). The patients were also evaluated at the ward. Group B patient’s experienced less pain compared to the other groups and the difference was statistically significant (Table 3). The mean highest scores of pain at ward were also compared between the groups A and B, groups C and A and groups C and B (Table 4). 

**Table 1.** The Comparison of Post-Surgical Pain Scores at PACU Between the Three Groups of Patients

	No.	Pain Score, Mean ± SD	P value
**Ibuprofen**	21	8.14 ± 0.85	0.001
**Acetaminophen**	18	7.05 ± 0.63	0.001
**Control**	21	8.36 ± 1.03	0.001

**Table 2.** The Comparison of Post-Surgical Pain Scores at PACU Between Each Two Groups of Patients

	Mean Differences	Standard Deviation	P value
**Ibuprofen-Acetaminophen**	1.08	0.30	0.002
**Ibuprofen-Control**	0.23	0.29	0.692
**Acetaminophen-Control**	- 1.32	0.30	0.001

**Table 3.** The Comparison of Post-Surgical Pain Scores at Ward Between the Three Groups of Patients

	No.	Pain Score, Mean ± SD	P value
**Ibuprofen**	21	7.75 ± 0.78	0.001
**Acetaminophen**	18	6.77 ± 0.64	0.001
**Control**	21	8.04 ± 1.02	0.001

**Table 4.** The Comparison of Post-Surgical Pain Scores at Ward Between Each Two Groups of Patients

	Mean Differences	Standard Deviation	P value
**Ibuprofen-Acetaminophen**	0.97	0.02	0.002
**Ibuprofen-Control**	-0.29	0.26	0.500
**Acetaminophen-Control**	- 1.26	0.27	0.001

## 5. Discussion

Opioids are rarely used for pain management after tonsillectomy due to their adverse side-effects, including nausea, vomiting and respiratory depression (20, 21).In this study we compared postoperative pain score following adenotonsillectomy in children given either acetaminophen ibuprofen or placebo, Based on the results the mean pain score at PACU and ward were significantly lower in patients who received acetaminophen before the operation, compared to the ibuprofen and control groups. However, there was no significant difference between patients receiving ibuprofen and control group. Young and colleagues demonstrated that premedication with acetaminophen reduced hydromorphone consumption and opioid-related side effect in patients undergoing abdominal hysterectomy, but did not significantly reduce the pain intensity (22). However, Bennie and colleagues demonstrated in their study that high dose of acetaminophen had similar analgesic effect as ibuprofen after myringotomy in pediatric patients. In addition, there was no significant difference between the analgesic effects of acetaminophen, ibuprofen and placebo after myringotomy (18). Bremerich and colleagues reported in their study that pre-operative rectal acetaminophen administration (10, 20, 40 mg/kg) was not effective in pain management after the operation; however, titrated IV opioid boluses produced rapid and reliable pain relief (23). In Bird's study was demonstrated that there was no significant difference between acetaminophen and ibuprofen's pain relief effects after separator placement (24). Primosch et al. reported in their study that preoperative acetaminophen or ibuprofen administration was not statistically superior to placebo administration to manage the post extraction pain (19). In a study colleagues concluded that acetaminophen administration (40 mg/kg) resulted in pain relief and CHEOPS < 9 in 87% of pediatric patients (16). In a study colleagues evaluated perioperative effects of oral ketorolac and acetaminophen in children undergoing bilateral myringotomy in their study and reported that 70% of cases who underwent myringotomy and tube placement required analgesia after the operation (17). In this study, postoperative pain after adenotonsillectomy was studied .The amount of tissue damage and tissue manipulation in the adenotonsillectomy is more significant than myringotomy. This causes more inflammation, increasing the drugs' effectiveness. The racial differences should also be considered. It is also possible that the differences in the observed effects of these drugs are due to the differences in their bioavailability. For example, Van der westhuizen concluded that Paracetamolexerts more therapeutic plasma concentration when administered intravenously (25). Kaluzny and colleagues reported that preoperative oral administration of 1 gr acetaminophen was effective, convenient, safe and cost effective in reducing the pain during and following the operation, in phacoemulsification performed using topical anesthesia (26). The administration time of these analgesics is another factor involved in the issue. Both acetaminophen and ibuprofen are absorbed rapidly from gastrointestinal tract when administered orally. Twenty seven to 60 minutes after acetaminophen absorption and 54 to 90 minutes after ibuprofen absorption, they reach to their peak plasma levels (27). In this study, drugs were administered 30 minutes before the operation; therefore, acetaminophen had reached its peak of plasma level but ibuprofen had not. The possibility of existence of the opioid-sparing effects of acetaminophen is also to be considered (23).

Based on the results of this study, it is concluded that administration of 15 mg/kg acetaminophen 30 minutes before the operation significantly decreases the CHEOPS score after adenotonsillectomy, in pediatric patients compared to 10 mg/kg ibuprofen. Further studies are recommended in addition to measurement of drugs plasma levels and evaluation of the effects of higher doses of the two studied analgesics. In addition, investigating other ways of administration of these analgesics is an interesting topic for future researches.
